# Comparison of INTREPID® balanced and hybrid tips on anterior capsule rupture in *ex vivo* porcine eyes

**DOI:** 10.1371/journal.pone.0290657

**Published:** 2023-08-29

**Authors:** Kei Ichikawa, Yoshiki Tanaka, Seiji Tokiwa, Airi Naito, Yuji Hidaka, Reiko Ichikawa, Kazuo Ichikawa, Naoki Yamamoto

**Affiliations:** 1 Chukyo Eye Clinic, Nagoya, Aichi, Japan; 2 General Aoyama Hospital, Toyokawa, Aichi, Japan; 3 Support Office for Bioresource Research, Center for Translational Research, Translational Research Headquarters, Fujita Health University, Toyoake, Aichi, Japan; 4 International Center for Cell and Gene Therapy, Research Promotion Headquarters, Fujita Health University, Toyoake, Aichi, Japan; St. Marianna University School of Medicine, JAPAN

## Abstract

Phacoemulsification has emerged as the global standard for cataract surgery, and various novel methods, tools, and agents have promoted surgical efficiency and reduced complications. Conventionally, the phaco tip, which cleaves and aspirates the cataractous lens, has been mainly constructed of metal. In this study, the risk of anterior capsule rupture was evaluated under conditions of different power modes, longitudinal (Mode-L), torsional (Mode-T), or both (Mode-LT), and different aspiration powers (0 or 200 mmHg), using a traditional metal phaco tip (Group-M) or a new phaco tip with a high-strength polymer overmold on the needle edge (Group-P), which was developed to reduce the risk of capsule rupture. One hundred twenty porcine eyes were used for experiments within a setting of typical human physiological intraocular pressure. We found that Group-M showed capsule rupture with a smaller ultrasound power than did Group-P, regardless of power mode or aspiration power. In Group-M, there was no significant difference in risk of capsule rupture among power modes, however in Group-P, capsule rupture was least likely to occur with Mode-T. These results provide useful information for inexperienced ophthalmologists to improve surgical safety.

## Introduction

The most frequent cause of reversible blindness globally is age-related cataract [1]. Phacoemulsification and aspiration was invented by Dr. Charles Kelman in 1967 [2] and is currently the primary method used in cataract surgery worldwide. A variety of innovative methods, tools including phaco tips and sleeves, and agents such as viscoelastic materials, have enhanced effectiveness, improved outcomes, and reduced complications in cataract surgery [3]. A cataractous lens is crushed using a tip attached to the needle edge of an ultrasound handpiece, and the fragments are aspirated with perfusate. The material of the tips used to be mostly metal, but a tip with a high-strength polymer overmold was recently developed to reduce the risk of posterior capsule rupture. Shumway *et al*. compared the novel polymetric tip with a conventional tip in cadaver eyes [4]. After lensectomy, the novel tip contacted the posterior capsule with the bevel up, the upper limit of aspiration power was set at 150 mmHg and the aspiration flow rate at 30 cc/min, then torsional mode (Mode-T) power was increased in steps of 5% to a maximum ultrasound power of 60%. The primary endpoint was the power of Mode-T required to cause posterior capsule rupture. In their study, ultrasound power was evaluated only in Mode-T. However, not only Mode-T but longitudinal (Mode-L), and a combination of both (Mode-LT) are used in cataract surgery. Nair *et al*. compared the mean temperatures of the shaft and tip in the eyes of 22 patients who underwent phacoemulsification and were divided into 6 groups by ultrasound power (Modes-L, T, or LT) and nuclear sclerosis grades (N2 or N3), resulting in the shaft having a higher mean temperature than the tip in Mode-T [5]. It is important to evaluate tips by ultrasound power modes utilized in clinical surgery. There has not been a study to date that compares risk of capsule rupture by tip material and ultrasound power settings. Here, we compared a metal vs a polymetric tip by risk of capsule rupture under the different conditions of longitudinal, torsional, and combination of both ultrasound power modes, and aspiration power.

## Materials and methods

### Eyes

Fresh porcine eyes (age about 5 to 6 months) brought from a local slaughterhouse with lids closed to avoid damage and exposure to the air were stored at 4°C until use by 10 hours after enucleation. Due to their similar morphology to the human eye, porcine eyes are often used for *ex vivo* animal models in vision sciences research [6–8]. They were washed with saline (Otsuka Pharmaceutical Factory, Inc., Tokyo, Japan) and inspected under a microscope (OPMI VISU 150, on S7 Stand, Carl Zeiss Meditec AG, Jena, Freistaat Thüringen, Germany) to exclude any with lacerations or perforations. One hundred twenty eyes with corneas of similar physical characteristics such as size to the human cornea (approx. 12 mm) [9–11] were involved for the experiment. This study was conducted in accordance with the ARVO Statement for the Use of Animals in Ophthalmic and Vision Research. The ethics committee of the Chukyo Eye Clinic (Nagoya, Aichi, Japan) ruled that approval was not required for the study since the porcine eyes were byproducts of the slaughter process and not harvested for this study.

### Equipment

An active-fluidics and torsional phacoemulsification device (CENTURION® VISION SYSTEM, Alcon Laboratories, Inc., Vernier-Geneva, Switzerland) was utilized in all experiments performed by one ophthalmologist experienced in cataract surgery at Chukyo Eye Clinic.

### Phacoemulsification tips

**Fig 1:**
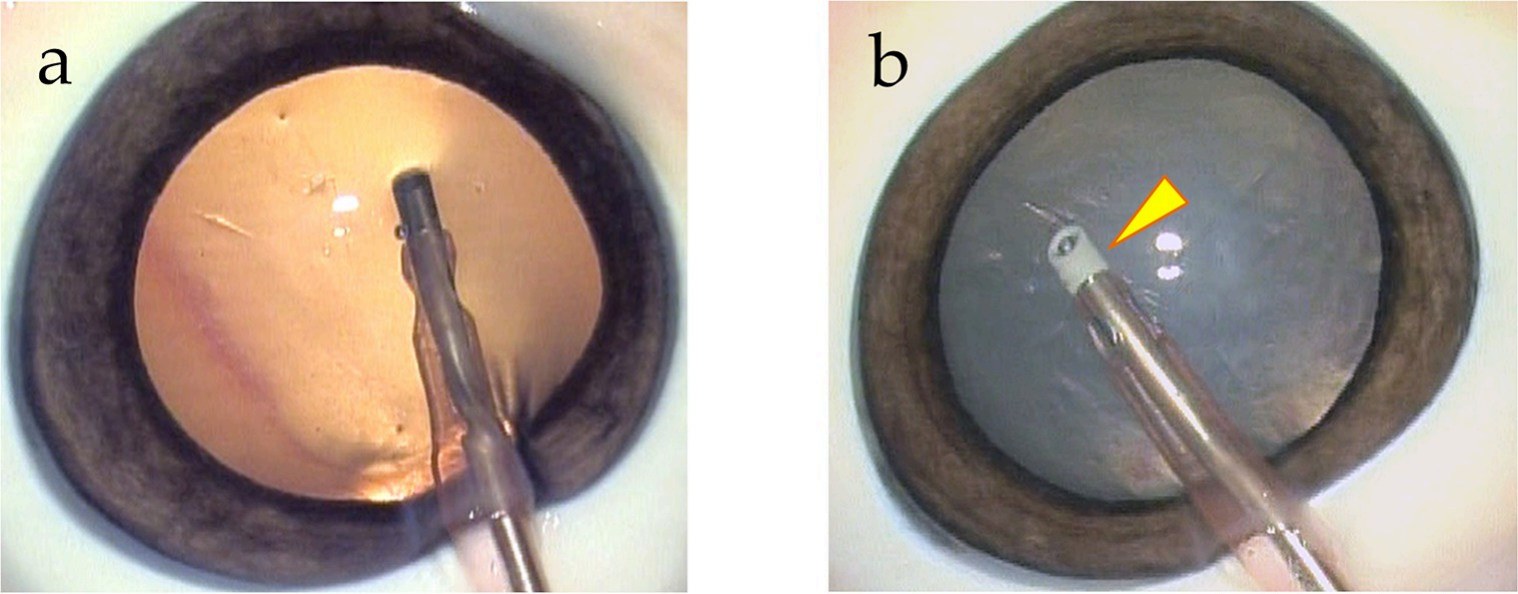
Two phacoemulsification tips pressed against the anterior capsule. (a) Metal INTREPID® Balanced Tip used in eyes of Group-M. (b) Polymeric INTREPID® Hybrid Tip used in eyes of Group-P. Both phaco tips applied ultrasound vibration to the anterior capsule of porcine eyes. The yellow arrowhead points to Group-P which has a high-strength polymer overmold.

Two phacoemulsification tips were compared (Fig 1); a metal INTREPID® Balanced Tip (Alcon Laboratories; Group-M), which minimizes movement near the incision wound but generates a large lateral amplitude at the tip, making it an efficient surgical tip; and an INTREPID® Hybrid Tip (Alcon Laboratories; Group-P), which has a rounded edge design with a polymeric material at the tip to reduce the risk of capsule rupture yet delivers the same ultrasound power. Both tips were used with the CENTURION® ACTIVE SENTRY® handpiece (Alcon Laboratories), which is equipped with a perfusion pressure sensor [12] that was not previously available on the handpiece, to maintain near-normal intraocular pressure.

### Experimental procedure

After making a corneal incision with a 3.2 mm Arc knife, the phaco tip was pressed against the anterior capsule with its bevel up. The device was set to maintain an intraocular pressure of 20 mmHg to replicate the human eye environment (normal intraocular pressure) [13, 14], and the aspiration flow rate was set at 30 cc/min. Under the two aspiration powers of 0 and 200 mmHg, the ultrasound power was set to 5% to check for presence of capsule rupture. If no rupture occurred, the ultrasound power was increased in increments of 5% to reach 100% with change in the position on the anterior capsule pressed by the tip. These steps were repeated using Mode-L, Mode-T, and Mode-LT, respectively, with the ultrasound power at time of any rupture recorded. The mean ultrasound power among increments one step before rupture was determined as the maximum value that did not cause rupture, hereafter referred to as threshold; if rupture did not occur ≤100% ultrasound power, the threshold value was calculated as 100%.

### Statistical analysis

Data presented as mean ± standard deviation (SD) were analyzed by Kruskal-Wallis followed by Scheffe’s post hoc test to compare among three or more groups of independent data, and Mann-Whitney U was used to compare among pairs of groups, using SPSS Statistics 24 (IBM Corporation, New York, NY, USA).

## Results and discussion

### Mean ultrasound power thresholds of capsule rupture

Table 1 shows the thresholds under different conditions. Higher values indicate less tendency to rupture at higher ultrasound powers. Each 10 eyes were used for each condition.

**Table 1:** Mean and standard deviation of thresholds of capsule rupture.

Tip type	Aspiration power	Longitudinal (n = 40)	Torsional (n = 40)	Combination (n = 40)
M	0 mmHg	32.0 ± 12.5	22.5 ± 4.2	20.5 ± 4.4
	200 mmHg	31.0 ± 6.1	23.5 ± 4.7	24.0 ± 5.2
	*p*-value	n.s.	n.s.	n.s.
P	0 mmHg	73.5 ± 7.5	98.0 ± 6.3	84.5 ± 5.0
	200 mmHg	55.5 ± 14.6	92.5 ± 7.9	71.5 ± 5.8
	*p*-value *	< 0.01	< 0.05	< 0.01

M, metal tip; P, polymeric tip

*significant difference (Mann-Whitney U test).

There was no significant difference in the thresholds between 0 and 200 mmHg aspiration power in Group-M regardless of mode type.

In contrast, in Group-P, capsules were more likely to rupture at 200 mmHg than 0 mmHg aspiration power. Among modes, Mode-L and Mode-LT significantly differed from Mode-T, which was less susceptible to capsule rupture at higher thresholds of ultrasound power since five capsules at 0 mmHg and two at 200 mmHg did not rupture under 100% force.

### Association between tip type and power mode at 0 mmHg aspiration power (Fig 2)

Capsules in Group-M eyes ruptured at significantly lower thresholds than in Group-P, regardless of power mode at 0 mmHg aspiration power. In Group-M, Mode-LT was most likely to cause rupture and Mode-L was least likely. In Group-P, Mode-L was most likely to cause rupture and Mode-T was least likely. Mode-T of Group-P showed the highest ultrasound power among the six groups yet was least likely to cause rupture, and was significantly higher than all modes in Group-M. Mode-LT in Group-P showed the next highest ultrasound power, and was significantly higher than Mode-T and Mode-LT in Group-M.

**Fig 2:**
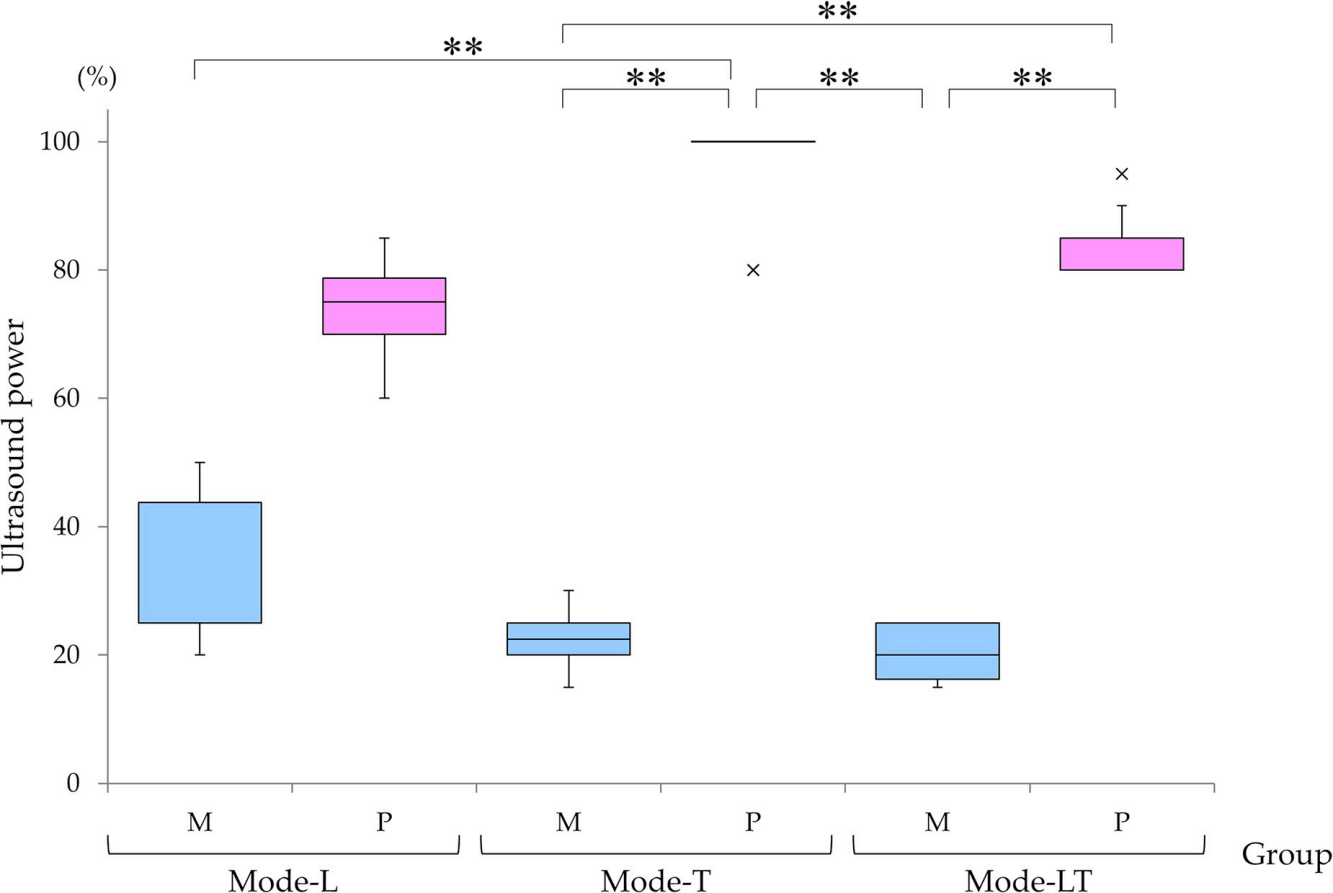
Ultrasound power by tip type and power mode at 0 mmHg aspiration power. At 0 mmHg aspiration, capsules in eyes treated with Mode-T in Group-P were the least likely to rupture even at the highest ultrasound power among the six groups; and power required to rupture capsules was significantly higher for Mode-T in Group-P than with any mode in Group-M. Next, the combination ultrasound Mode-LT in Group-P could effectively operate without capsule rupture with significantly higher power than Mode-T and Mode-LT in Group-M. *P*-value was calculated using Kruskal-Wallis followed by Scheffe’s post hoc test; **: *p* < 0.001.

### Association between tip type and power mode at 200 mmHg aspiration power (Fig 3)

Capsules in Group-M eyes ruptured at significantly lower power than those in Group-P regardless of mode. Change in aspiration power did not significantly affect the results. Mode-T in Group-P showed the highest ultrasound power among the six groups with operated eyes least susceptible to rupture, and could achieve a significantly higher non-rupture force than all modes of Group-M. Mode-LT in Group-P showed the next highest ultrasound power, which was significantly higher than Mode-T and Mode-LT in Group-M. These findings were similar at both aspiration powers, 0 mmHg and 200 mmHg.

**Fig 3:**
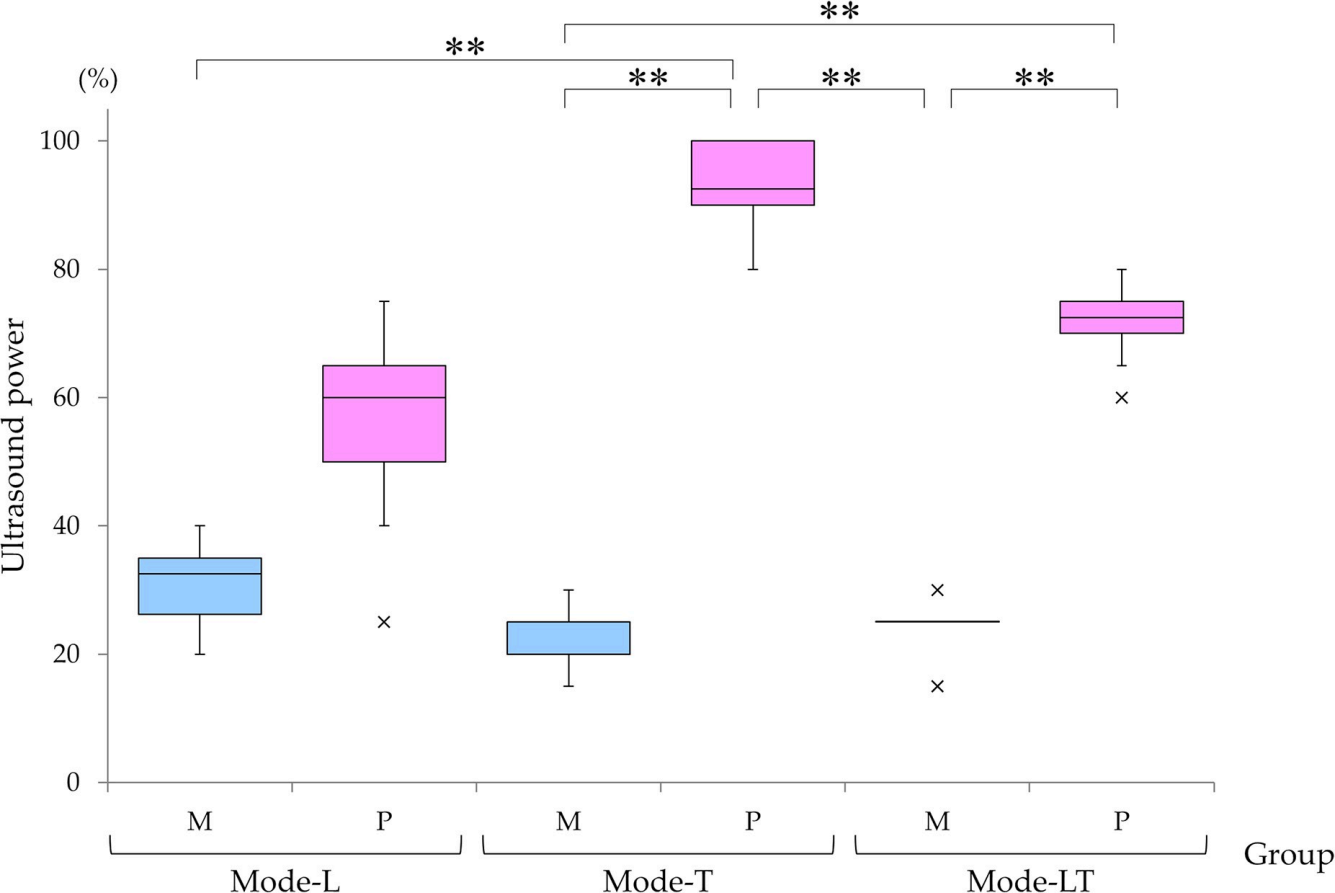
Ultrasound power by tip type and power mode at 200 mmHg aspiration power. At 200 mmHg aspiration, capsules in eyes treated with Mode-T in Group-P were the least likely to rupture even at the highest ultrasound power among the six groups; and power required to rupture capsules was significantly higher for Mode-T in Group-P than with any mode in Group-M. Next, the combination ultrasound Mode-LT in Group-P could effectively operate without capsule rupture with significantly higher power than Mode-T and Mode-LT in Group-M. These findings were similar to those at 0 mmHg. *P*-values were calculated using Kruskal-Wallis followed by Scheffe’s post hoc test; **: *p* < 0.001.

In the present study, the anterior capsule in eyes treated with the metal phaco tip ruptured at lower ultrasound power than did that in eyes treated with the high-strength polymer cover tip regardless of ultrasound power mode type, namely longitudinal, torsional, or a combination of both. Thus, the power mode impact on capsule rupture depended on tip type.

It has been reported that recent cataract surgery requires less energy to emulsify opacified lenses as previously, since the number of cases presenting with high grades and hard nuclei has decreased [15–23]. Also, besides removing opacified lenses one can significantly reduce the risk of postoperative corneal astigmatism with smaller incision sizes and sophisticated intraocular lenses, resulting in good postoperative visual acuity through refractive correction [24]. More efficient energy transmission to the phaco tip was reported for Group-M than Kelman tips [25]; and safer crushing and aspiration of lens cortex and nucleus due to the polymer overmold of a Group-P tip avoided damage to the iris and lens capsule [24]. There have been few reports of the polymer overmold tip; one compared efficacy and safety among the Active Sentry handpiece with Group-P and the Centurion OZil handpiece with Group-M in clinical surgical data, and reported the former showed significantly lower cumulative dissipated energy and torsional amplitude with no cataract surgery complications observed [24].

In another experiment two tips (30°, 0.9 mm), namely Kelman (standard tip) vs. OZil INTREPID® Balanced tip (torsional tip), were compared in porcine eyes, The lens nuclei was fixed in formalin and diced into cubes sized 2.0 mm. The latter tip type was much more efficient than the former throughout the range tested taking 29% less time to remove lens fragments (*p* < 0.0001), and 100% torsional power was 45% more efficient than 60% torsional power (*p* = 0.0028) [26]. At 100% longitudinal power, when the tip is moving forwards physically interacting with the lens material, the latter experienced more chatter, which means incidents when the lens material bounced off the tip during forward motion, under 60% (*p* = 0.001) and 100% torsional power (*p* = 0.0022). There was also some chatter at 75% longitudinal motion, but apart from these, overall it was practically absent. These results indicate that torsional tips crush the lens nucleus more efficiently than standard tips.

Using the same method, Ronquillo *et al*. investigated optimal longitudinal power settings for Infiniti OZil Intelligent Phaco at varying torsional amplitude settings with lens cube phacoemulsification performed at 60%, 80%, and 100% torsional amplitude and 0% to 100% longitudinal power. They reported efficiency in crushing lens nucleus cubes significantly increased at 60% and 80% torsional amplitude with increasing longitudinal power [27].

In the present study, the same handpiece was used, and all other conditions were the same but aspiration power (0 or 200 mmHg) to compare performance between tips (with or without a polymer overmold) under different power modes (longitudinal, torsional, or longitudinal + torsional). In Group-M, there was no significant difference in mean ultrasound power threshold required for anterior capsule rupture by power mode between 0 and 200 mmHg aspiration power. Since anterior capsules ruptured in Group-M at lower ultrasound power than in Group-P, it is assumed the lack of polymetric material on the needle edge affected aspiration power at 0 and 200 mmHg. In contrast, in Group-P, the mean ultrasound power threshold was significantly lower at 200 mmHg than 0 mmHg in all modes (*p* < 0.05) (S1 Table). This suggests the polymer overmold made the anterior capsule less likely to rupture compared to Group-M, but slightly more likely to rupture at 200 mmHg.

Since Mode-L crushes with one cut per stroke and aspirates coaxially, the lens nucleus is easily repelled, reducing retention of the nucleus and surgical efficiency. In contrast, Mode-T crushes with two cuts per stroke and aspirates on different axes and the lens nucleus is not repelled, resulting in superior retention of the nucleus and good surgical efficiency. Mode-LT produced both of those effects.

**Fig 4:**
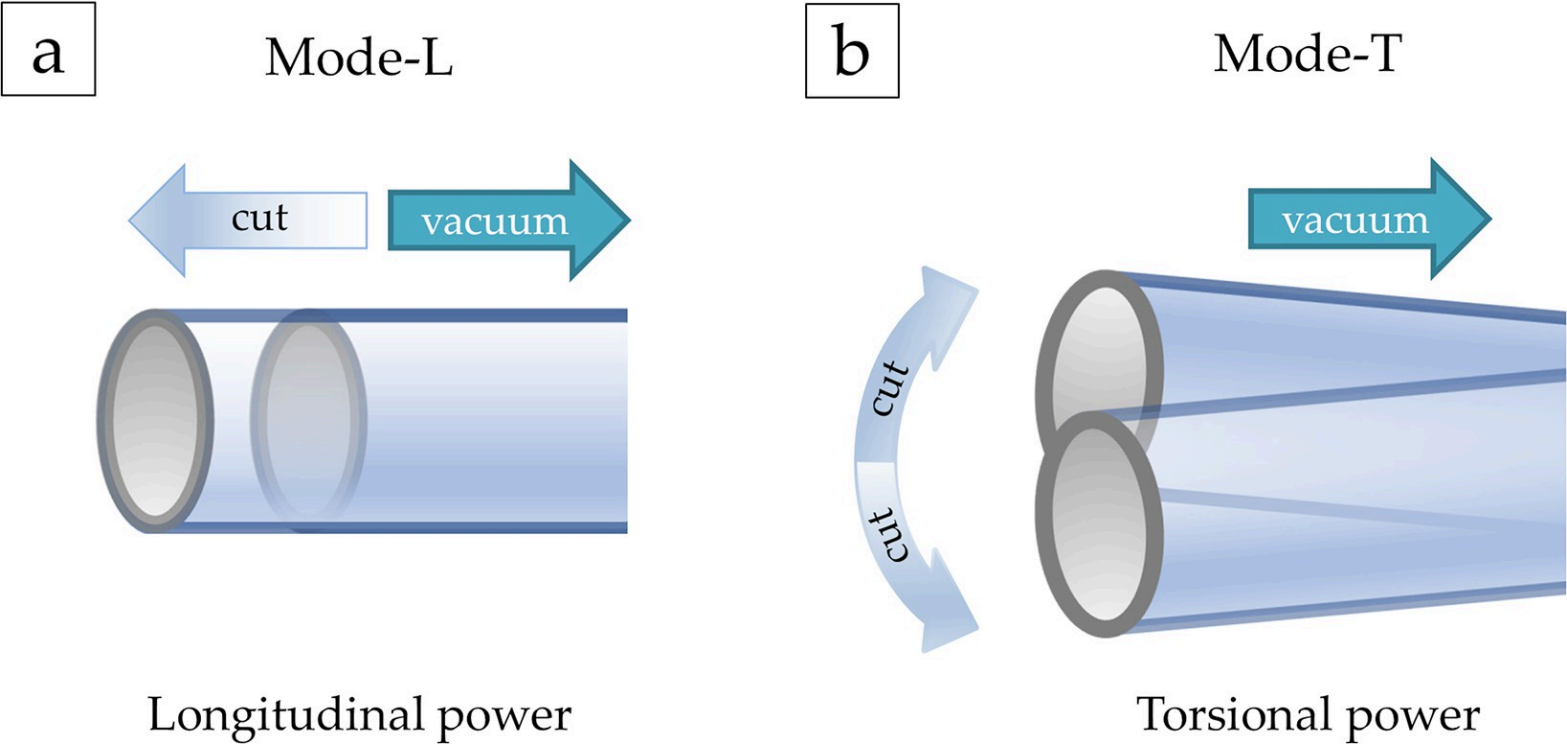
Direction of amplitude and aspiration in Mode-L and Mode-T. (a) In longitudinal mode, the phaco tip performs one cut of crushing per stroke. Because aspiration and crushing are performed on the same axis, the lens nucleus is easily played, resulting in reduced retention and efficiency of the lens nucleus. (b) In torsional mode, the phaco tip performs two cuts of crushing per stroke. Because aspiration and crushing are performed on different axes and do not rupture the lens nucleus, it has superior retention and efficiency of the lens nucleus.

In Group-P, albeit not significant, there were differences in ultrasound power among power modes. The order the anterior capsule was least likely to rupture even with high ultrasound power was Mode-T, Mode-LT, and Mode-L, respectively. It was assumed that the greater the longitudinal factor of the power mode, the more likely the anterior capsule would rupture even with low ultrasound power (Fig 4).

In this study, fresh lens-free porcine eyes, aged between 5 to 6 months, were used. The experiment was conducted 10 times under 12 different conditions, requiring a total of 120 lenses that were prepared to ensure reproducibility. Being morphologically similar to human eyes, porcine eyes are commonly used in vision sciences research [6–8]. The thickness of the porcine lens anterior chambers used in the experiments ranged from 50 to 66 μm (median 59 μm), and previous studies have reported good reproducibility in biomechanical measurements of porcine lenses [28]. In contrast, it was reported that the thickness of the lens capsule in humans aged 12 to 103 years old increased from an average of 11 μm at the anterior pole to an average of 15 μm with age. The thickest part of the lens capsule was in the anterior midperiphery region, where it increased from an average of 13.5 μm to an average of 16 μm with age. The equatorial area averaged 7 μm, and the posterior pole was the thinnest, averaging 3.5 μm. The posterior pole was reported to become thinner with age, decreasing from an average of 9 μm to an average of 4 μm [29]. It was also reported that the porcine lens capsule was approx. 4-fold thicker than the human lens capsule, but 50% less rigid [30]. These differences in mechanical properties, such as thickness and stiffness, are expected to reflect differences in the structure of the lens capsule. Observations using atomic force microscopy revealed that the interfibrillar spacing in the porcine lens capsule measured 0.68 ± 0.25 μm, whereas in the human lens capsule it measured 1.08 ± 0.25 μm. The surface of the porcine lens capsule appeared smooth, while the human lens capsule was much rougher. The interfibrillar spacing in human lens capsules decreased with age. On the other hand, the diameter of the fibers comprising the lens capsules of porcine averaged 306 ± 239 nm (range: 50 to 950 nm), whereas they averaged 339 ± 135 nm (range: 67 to 691 nm) in humans [31]. There was no significant difference in fiber thickness between porcine and human lenses, suggesting that the difference in lens capsule thickness was dependent on interfibrillar spacing. In this study, although there were differences in the thickness and stiffness of porcine lens capsules compared to human lens capsules, porcine lenses were used to ensure experimental reproducibility and uniformity of experimental conditions. However, since the thickness of the fibers forming the lens capsules does not significantly differ between the two types of lenses, we believe that the findings of this study may be extrapolated to understand the effects on the lens capsule during human cataract surgery.

This study has some limitations. First, we used the anterior capsule of porcine lenses instead of the posterior capsule of human lenses. There is a method of removing the vitreous by making an incision in the equatorial part of the eye and placing a tip on the posterior pole for examination, but it is not possible to aspirate with constant pressure because it becomes a release space. And, since the anterior capsule is slightly thicker than the posterior capsule, it was assumed the posterior capsule could be ruptured with a slightly lower ultrasound power [28, 32–34]. Second, pig transparent lenses were used in this study. To the extent that aged human lenses, which have gradually become cloudy and hardened over the years, may respond differently to the tip, the tip efficiency cannot be fully predicted in this study [12]. Third, the effect of heat generated at the tip on capsule rupture may have been greater than in human eyes since no viscoelastic material was present in the non-cataractous porcine lenses [35, 36]. Fourth, the results pertain to a single physician. Performance may vary between physicians. However, the report is considered to contain useful information and advice for novice surgeons to improve safety. We aim to determine clinically useful ultrasound parameters in experiments with cadaver eyes in the future.

## Conclusions

Regardless of ultrasound setting, the polymer overmold tip significantly reduced the risk of capsule rupture compared to the metal tip. The polymer overmold tip with torsional ultrasound power showed the lowest risk of capsule rupture.

## Supporting information

S1 Table Experimental results for mean threshold and standard deviation of maximum ultrasound power that did not cause capsule rupture. (XLSX)
